# Supplementary material for: Estimate of Growth Parameters of Penaeus kerathurus (Forskäl, 1775) (Crustacea, Penaeidae) in the Northern Adriatic Sea
Source: Animals (Basel). 2024 Mar 31;14(7):1068. doi: 10.3390/ani14071068 (PMC11010835; doi:10.3390/ani14071068)
Supplement: Supplementary file 1 [file animals-14-01068-s001.zip › animals-2857329-supplementary.pdf]

Supplementary Material

Estimate of growth parameters of *Penaeus kerathurus* (Forskäl, 1775) (Crustacea, Penaeidae) in the North Adriatic Sea

Martina Scanu, Carlo Froglia, Fabio Grati and Luca Bolognini

For the production of the following Figures, growth parameter values for Linf, K and t_a were averaged between the 2 algorithms and the 2 VBGF configurations used, as presented in Table 3 of the Manuscript. On the other hand, the specific seasonal values (t_s and C) were simply averaged between the 2 algorithms (ELEFAN_GA_boot and ELEFAN_SA_boot).

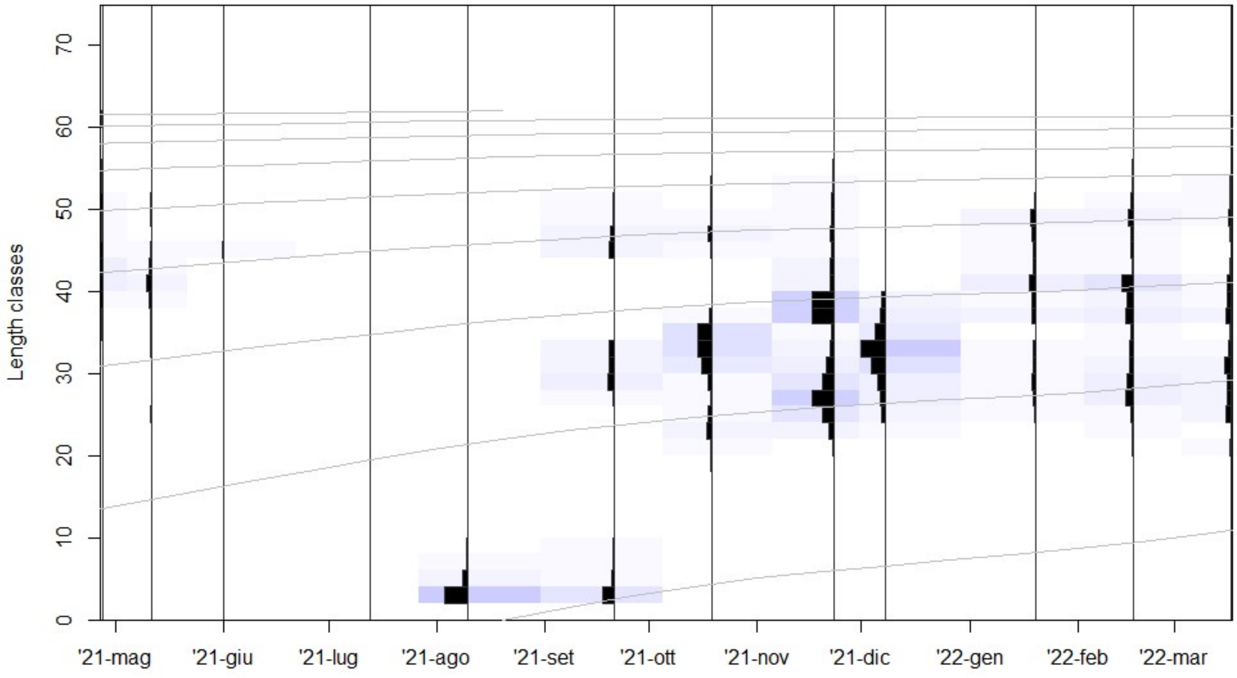

Figure S1: Monthly LFDs of Female individuals together with the fitting of the averaged seasonal parameters.

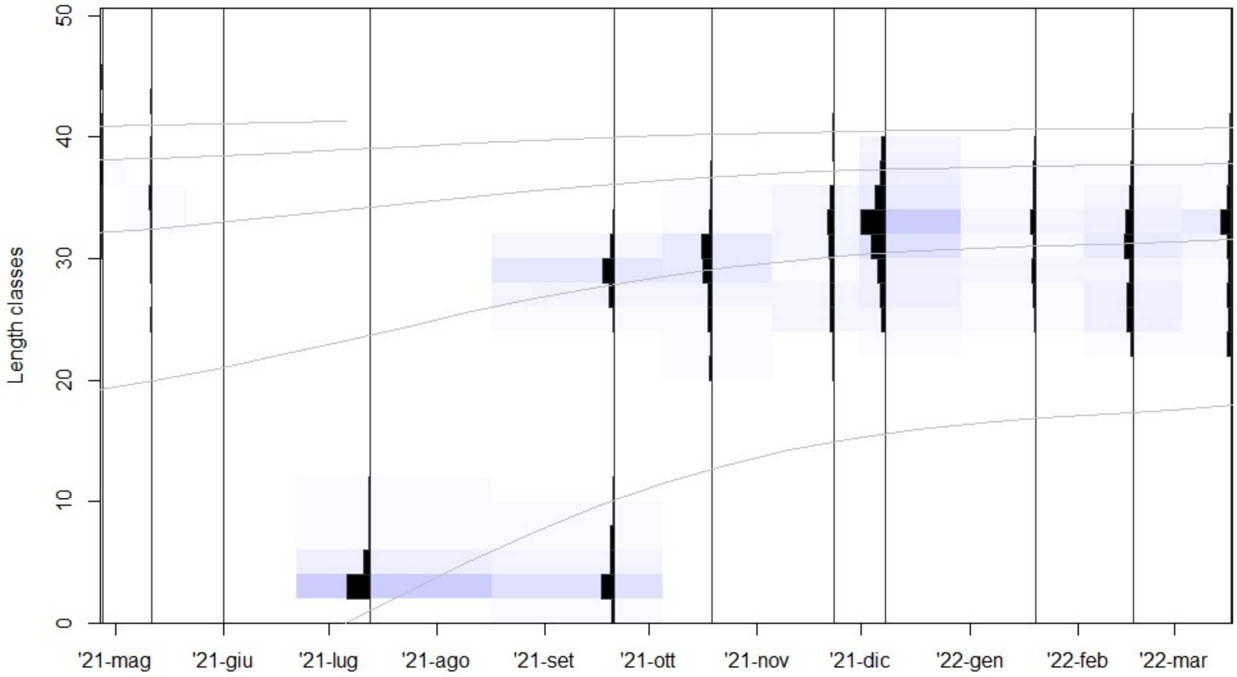

Figure S2: Monthly LFDs of Male individuals together with the fitting of the averaged seasonal parameters.
